# Sodium glucose transporter 2 (SGLT2) inhibition with empagliflozin improves cardiac diastolic function in a female rodent model of diabetes

**DOI:** 10.1186/s12933-016-0489-z

**Published:** 2017-01-13

**Authors:** Javad Habibi, Annayya R. Aroor, James R. Sowers, Guanghong Jia, Melvin R. Hayden, Mona Garro, Brady Barron, Eric Mayoux, R. Scott Rector, Adam Whaley-Connell, Vincent G. DeMarco

**Affiliations:** 1 Department of Medicine, Division of Endocrinology, Diabetes and Cardiovascular Center, University of Missouri, School of Medicine, Columbia, USA; 2 Division of Nephrology, University of Missouri, School of Medicine, Columbia, USA; 3 Department of Medical Pharmacology and Physiology, University of Missouri, School of Medicine, Columbia, USA; 4 Research Service, Harry S. Truman Memorial Veterans Hospital, Columbia, MO USA; 5 The Dalton Cardiovascular Research Center, Columbia, MO USA; 6 Departments of Medicine-Gastroenterology and Hepatology and Nutrition and Exercise Physiology, University of Missouri, Columbia, MO USA; 7 Department of Cardiometabolic Diseases Research, Boehringer-Ingelheim, Biberach, Germany

**Keywords:** Empagliflozin, SGLT2 inhibitor, Diastolic function

## Abstract

Obese and diabetic individuals are at increased risk for impairments in diastolic relaxation and heart failure with preserved ejection fraction. The impairments in diastolic relaxation are especially pronounced in obese and diabetic women and predict future cardiovascular disease (CVD) events in this population. Recent clinical data suggest sodium glucose transporter-2 (SGLT2) inhibition reduces CVD events in diabetic individuals, but the mechanisms of this CVD protection are unknown. To determine whether targeting SGLT2 improves diastolic relaxation, we utilized empagliflozin (EMPA) in female db/db mice. Eleven week old female db/db mice were fed normal mouse chow, with or without EMPA, for 5 weeks. Blood pressure (BP), HbA1c and fasting glucose were significantly increased in untreated db/db mice (DbC) (P < 0.01). EMPA treatment (DbE) improved glycemic indices (P < 0.05), but not BP (P > 0.05). At baseline, DbC and DbE had already established impaired diastolic relaxation as indicated by impaired septal wall motion (>tissue Doppler derived E′/A′ ratio) and increased left ventricular (LV) filling pressure (<E/E′ ratio). Although these abnormalities persisted throughout the study period in DbC, diastolic function improved with EMPA treatment. In DbC, myocardial fibrosis was accompanied by increased expression of profibrotic/prohypertrophic proteins, serum/glucocorticoid regulated kinase 1 (SGK1) and the epithelial sodium channel (ENaC), and the development of these abnormalities were reduced with EMPA. DbC exhibited eccentric LV hypertrophy that was slightly improved by EMPA, indicated by a reduction in cardiomyocyte cross sectional area. In summary, EMPA improved glycemic indices along with diastolic relaxation, as well as SGK1/ENaC profibrosis signaling and associated interstitial fibrosis, all of which occurred in the absence of any changes in BP.

**Electronic supplementary material:**

The online version of this article (doi:10.1186/s12933-016-0489-z) contains supplementary material, which is available to authorized users.

## Background

The global epidemic of obesity is largely responsible for the increased incidence of type 2 diabetes mellitus and associated cardiovascular diseases (CVD). Affected individuals are likely to have one or more CVD complications, such as hypertension and cardiac diastolic dysfunction; indeed, diabetes is often considered a CVD equivalent. In this regard, young (premenopausal) obese and diabetic women are particularly vulnerable to CVD [1, 2]. Normally, young lean woman are at lower risk for development of CVD compared to men. However, this sex-related cardioprotection is lost in conditions of obesity or diabetes [3]. Diastolic dysfunction, which is defined by delayed diastolic relaxation [4], is associated with insulin resistance, cardiac fibrosis, hypertrophy, and inflammation [5, 6]. Diastolic heart failure, a CVD risk factor usually associated with aging, has dramatically increased in incidence in association with the increases in obesity and diabetes. Therefore, developing new strategies to improve both glycemia and CVD out comes in individuals afflicted with diabetes would be highly desirable, especially for women.

Diastolic dysfunction is typically associated with interstitial fibrosis and left ventricular (LV) hypertrophy that promote LV stiffness and impaired relaxation. In this regard, serum and glucocorticoid regulated kinase 1 (SGK1), which is highly expressed in the diabetic heart and is stimulated by excess circulating glucose, is emerging as a mediator of cardiac fibrosis/stiffening and impaired cardiac relaxation [7–10]. SGK1 stimulates a number of ion channels, including the epithelial sodium channel (ENaC), as well as transporters, transcription factors and enzymes [11]. Indeed, we recently reported that fibrosis and stiffening of the aorta of overweight female mice fed a high fat, high sugar diet for 4 months was associated with increased aortic expression of SGK1 and ENaC [12].

In diabetes, hyperglycemia promotes a state of glucotoxicity, inflammation and oxidative stress which are associated with hypertension and end organ injury, including injury to the heart. In this regard, pharmacologic inhibitors of the renal sodium–glucose cotransporter 2 (SGLT2) are emerging as a novel group of drugs that lower blood glucose and HbA1c levels and improve whole body insulin sensitivity in animals and humans with diabetes, largely by blocking renal proximal tubular reabsorption of glucose which increases urinary glucose excretion (glycosuria) [13, 14]. The mild osmotic effects of SGLT2 inhibitor treatment can lead to modest reduction in blood pressure (BP), an effect, along with the improvement in glucose control and weight reduction that could reduce the risk of CVD. Indeed, a recent meta-analysis demonstrated favorable cardiovascular outcomes in diabetes patients treated with empagliflozin (EMPA) [15]. Administration of SGLT2 inhibitors to animals or humans with diabetes may also reduce adiposity, oxidative stress and expression of advanced glycation end products (AGE) and receptors for AGE (RAGE) [16]. Herein, we tested whether SGLT2 inhibitor therapy would attenuate the development of the earliest manifestation of diabetic heart disease, diastolic dysfunction, in part, by reducing blood pressure (BP), cardiac oxidative stress and pro-fibrotic factors. Diastolic dysfunction is especially pronounced in obese, insulin resistant and diabetic females [1, 2, 17–19]. Specifically, we hypothesized that the SGLT2i, EMPA would blunt the development/progression of diastolic dysfunction and the associated abnormalities in cardiac remodeling in insulin resistant female diabetic db/db mice (Lepr^db/db^). Previous reports demonstrate that female db/db mice develop diastolic dysfunction, cardiac fibrosis and left ventricular hypertrophy (LVH) [20–22]. The db/db model is clinically relevant in that hyperleptinemia and leptin resistance, obesity and associated heart disease are seen in the human obese population and leptin levels are elevated in conditions of chronic heart failure and chronic hypertension. The db/db mouse exhibits a non-dipping BP pattern, diastolic dysfunction and cardiac remodeling; these CVD features of metabolic disease are also observed in obese and insulin resistant humans [20, 23–27]. Herein, we examined whether the anticipated improvement in diastolic function and cardiac remodeling with EMPA treatment would be associated with reductions in myocardial interstitial fibrosis, profibrotic signaling proteins, oxidative stress and improvements in myocardial mitochondrial ultrastructure.

## Methods

### Animals

All animal procedures were approved by the Harry S Truman Veterans Affairs Memorial Hospital Subcommittee for Animal Safety (SAS) and the University of Missouri IACUC. Eight week old female db/db (BKS.Cg-Dock7^m^+/+Lepr^db^/J) and wild-type control (C57BLKS/J) mice were purchased from Jackson Labs and were housed under standard laboratory conditions where room temperature was 21–22 °C and light and dark cycles were 12 h each. Three different cohorts of mice were used for these studies. Each cohort consisted of three groups of mice including lean untreated controls (CkC), untreated db/db (DbC) and db/db treated with EMPA (DbE) for 5 weeks. In total there were 17 CkC, 19 DbC and 19 DbE. The first and second cohorts consisted of 5–6 mice per group and subsets of these mice were used for cardiac function, urine and plasma biochemistry, histological (light and electron microscopy) and immunological studies. The third cohort consisted of 6–7 mice per group that were used primarily for ambulatory BP monitoring. Two DbC and two DbE were removed from Cohort3 following acute complications from telemetry implant surgery. The radiotransmitter in another DbC failed in the middle of the study and one CkC with a radiotransmitter succumbed late in the study. Of the 55 mice used, six mice, all from Cohort3, did not complete the study. The treatment period began when mice were 11 weeks of age and ended 5 weeks later when mice were 16 weeks of age. Previous reports indicate that cardiac dysfunction and remodeling in female db/db mice begin to develop and progress during the time frame of our experiment [20–22].

### EMPA administration

EMPA is a potent and competitive inhibitor of SGLT2 with the highest selectivity profile of recently tested SGLT2i [28]. We mixed EMPA in normal mouse chow (Purina Diet 5008) at a concentration(60 mg kg^−1^ of diet) calculated to deliver 10 mg kg^−1^ day^−1^ based on food intake [13]. This dose of EMPA significantly improves HbA1c, 2 h glucose concentration during oral glucose tolerance test (OGTT) and insulin sensitivity by insulin euglycemic-hyperinsulinemic clamp and tends to lower circulating lipids in 17 week old female db/db mice [13].

### Blood and tissue biochemistry

Mice were fasted for 4 h in the morning (5–9 a.m.) prior to collection of a small blood sample from the tail vein. Blood was analyzed for glucose using an Alpha Trak II glucometer and for HbA1c using a DCA Vantage Analyzer (Seimens, Malvern, PA). Samples were drawn immediately prior to the start of the treatment period, midway and at the end of the study. At the end of the study mice were anesthetized with 3% isoflurane and a terminal blood sample was drawn immediately from the left ventricle. Blood was centrifuged and plasma was stored at −80 °C. Plasma was analyzed for insulin concentration using an ELISA kit specific for mouse insulin using a previously established protocol [29], as well as cholesterol, triglycerides, alanine aminotransferase and uric acid using commercially-available automated assays (Beckman-Coulter, Inc., Brey, CA). Liver samples were analyzed for triglyceride content using a previously established protocol [30]. Plasma electrolytes (Na^+^, K^+^ and Cl^−^) were measured on the same clinical chemistry platform using ion-specific electrodes.

### Urine analyses

Immediately prior to the start of treatment, in the middle of the treatment period and within 48 h of the end of treatment, mice were placed in metabolic chambers for 24 h urine collection and subsequent determination of concentrations of electrolytes and glucose. Urine was stored at −80 °C except for a 60 µl aliquot that was refrigerated overnight and analyzed for microalbumin and creatine, as well as the ratio of micro-albumin to creatine as a standardized measure of microalbuminuria, using a DCA Vantage analyzer.

### Body composition

Prior to both the start of treatment and the end of the study,whole body composition was analyzed by nuclear magnetic resonance spectroscopy (Echo MRI, Houston, TX).

### BP monitoring

Ambulatory BP and BP dipping status during the light and dark cycles were monitored weekly by radiotelemetry as previously described [31, 32].

### Echocardiography

Transthoracic echocardiography (TTE) was performed on isoflurane (1.75% in an oxygen stream) anesthetized mice as previously described [31, 33] using a GE Vividi ultrasound system. Initially, we performed TEE immediately prior to the beginning of the treatment period and again at the end of the treatment period.

### Quantification of interstitial fibrosis

One mm thick coronal slices of the heart just below the level of the papillary muscle were fixed in paraformaldehyde, embedded in paraffin, sectioned at 5 μ and stained for collagens using picro-Sirius-red, as previously described [34]. Images were taken with 5× and 40× objective lenses. A region of interest rectangle of known area was randomly placed in three areas and images were captured using a Nikon50i microscope, a 40× objective and cool snap*cf* camera and software. Each image was auto-leveled with Photoshop and the intensity of pink color was normalized to area and quantified using MetaVue software. For each animal an average estimate of fibrosis was calculated from the three determinations of collagen staining.

### Heart immunohistochemistry and immunoblotting

To evaluate the level of oxidative/nitrosative damage to myocardial proteins samples of the left ventricle (LV) free wall were fixed, embedded in paraplast, sectioned and assessed for 3-nitrotyrosine (3-NTY) residue using an immunofluorescence technique we described previously [34, 35]. LV tissue sections were also evaluated by immunofluorescence for wheat germ agglutinin (WGA, 1:50, #W11261, Invitrogen), AGE (1:100, #23722, Abcam), RAGE (1:50, #AF1179, R&D), SGK1 (1:100, #32374, Abcam) and ENaC (1:200, #77385, Abcam) expression. Average grey scale intensity was quantified within fixed region of interest rectangles as previously described [35].

Preparation of LV homogenates, electrophoresis and immunoblotting were described previously [34]. The following antibodies were used: phospho-Akt ^Ser308^ (1:1000; #4056, Cell Signaling Technology, Inc or CST), phospho-Akt ^Ser473^ (1:1000; #9271, CST), Akt (1:1000; #9272, CST), eNaC (1:1000; #77385, Abcam), RAGE (1:1000; #AF1179, R&D), Phospho-ERK ^T202/204^ (1:1000; #9106, CST), ERK (1:1000; #4695, CST) and pan-actin (1:1000; #4968, CST).

### Ultrastructure analysis using transmission electron microscopy (TEM)

Briefly, samples of the LV free wall were cut into 2 mm squares and placed immediately in primary TEM fixative as previously described. Specimens were then placed in resin and polymerized at 60 °C for 24 h. Ultrathin sections (85 nm) were stained with 5% uranyl acetate and Sato’s Triple lead stain. A JOEL 1400-EX TEM (Joel, Tokyo, Japan) was used to view three fields randomly chosen per mouse to obtain three 2000× images per LV [36].

### Statistical analysis

Results are reported as the mean ± SE. Differences in outcomes were determined using one-way ANOVA and Bonferroni post hoc tests for paired comparisons and were considered significant when P < 0.05. For comparisons regarding plasma insulin concentration at the end of the study, a Kruskal–Wallis ANOVA was used in place of standard ANOVA. All statistical analyses were performed using Sigma Plot (version 12) software (Systat Software, Point Richmond, CA).

## Results

### Baseline parameters

Compared to CkC mice, body weights of DbC and DbE mice were both 2.74-fold greater and this was due largely to the more than 12-fold increases in whole body fat mass, and to a lesser extent to 20 and 24% increases in whole body lean mass, in DbC and DbE, respectively (Additional file 1: Figure S1A; Table 1; P < 0.001). The slightly greater gain in lean mass in DbE compared to DbC did not reach statistical significance (Additional file 1: Figure S1B–E). The percentage of whole body water weight of DbC and DbE was less than half that of CkC and did not differ between the treated and untreated db/db groups.

**Table 1:** Baseline parameters, including body and liver weights, as well as post-treatment plasma metabolic markers of lean control (CkC), untreated db/db (DbC) and db/db mice treated with EMPA (DbE)

Parameter	ANOVA	CkC	DbC	DbE
	P value	(11)	(12)	(13)
Body weight				
Pre-treatment body weight (g)	0.001	18.9 ± 0.2	46.1* ± 0.7	45.4^§^ ± 0.7
Post-treatment body weight (g)	0.001	19.2 ± 0.3	53.6* ± 1.0	53.5^§^ ± 0.7
Delta (g)	0.001	0.17 ± 0.29	7.50* ± 0.76	8.32^§^ ± 0.50
% Increase in body weight	0.001	0.7 ± 1.6	13.8* ± 1.3	15.5^§^ ± 0.9
Hepatic				
Liver weight (mg)	0.001	835 ± 37	2460*^,†^ ± 92	2031^§^ ± 72
Triglycerides (nmol g^−1^)	0.001	15 ± 3	43* ± 5	37^§^ ± 4
Plasma				
Cholesterol (mg dl^−1^)	0.001	76 ± 5	145* ± 3	148^§^ ± 6
Triglycerides (mg dl^−1^)	0.001	126 ± 9	310* ± 18	301^§^ ± 20
Alanine aminotransferase (U l^−1^)	0.001	27 ± 2	78* ± 5	69^§^ ± 8

Values are mean ± SE. Post-hoc comparisons; P < 0.05 for * CkC vs DbC; ^†^ DbCvs DbE; and ^§^ CkC vs DbE. Sample sizes are noted in parentheses

### Metabolic parameters

Plasma cholesterol, triglycerides and alanine aminotransferase (ALT) were similarly elevated in both DbC and DbE indicating metabolic dyslipidemia and liver impairment in DbC and DbE (Table 1). Compared to CkC, liver triglyceride content was elevated in DbC and this was not altered by EMPA (Table 1). Plasma electrolytes did not differ among groups (not shown).

**Fig. 1:**
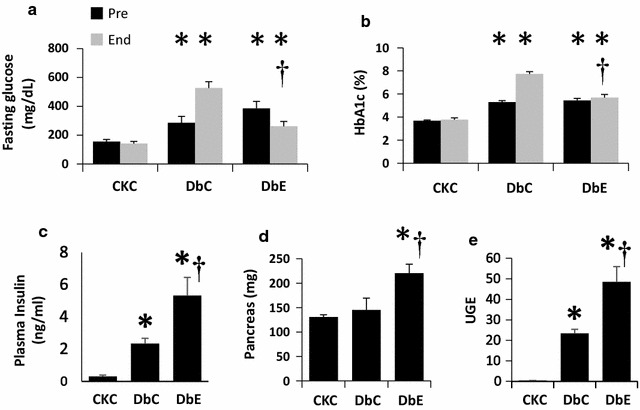
Empagliflozin improves dysglycemia in db/db mice. Db/db mice have elevated fasting glucose and HbA1c levels prior to the start of treatment. During the 5 week study period, dysglycemia was sustained in untreated db/db mice (DbC); however both **a** fasting glucose and **b** HbA1c were reduced in db/db treated with empagliflozin (DbE) by the end of the study. At the end of the study, DbE had increased **c** serum insulin concentrations and **d** pancreas mass **e** compared to CKC and DbC. **e** Compared to CkC, urine glucose excretion (UGE) was elevated in both db/db groups of mice, but UGE was twofold higher in DbE compared to DbC. *P < 0.05 compared to CkC at the same time point; ^†^P < 0.05 compared to DbC at the same time point

Baseline fasting glucose and HbA1c of CkC mice were 155 ± 15 mg dl^−1^ and 3.7 ± 0.1%, respectively and these parameters did not differ at the end of the study (Fig. 1a, b). At both measuring times, i.e., before treatment began and at the end of the treatment period, DbC and DbE mice had elevated fasting glucose and HbA1c consistent with diabetes, compared to CkC. By the end of the 5 week study period, the fasting glucose and HbA1c of DbC were both elevated compared to pre-treatment levels. Compared to pre-treatment values, DbE exhibited lower fasting glucose and HbA1c was unchanged. Moreover, both glycemic parameters in DbE were significantly reduced by the end of the treatment period compared to DbC. These data indicate that progressing hyperglycemia was abrogated by EMPA. In this study, 16 week old DbC and DbE had higher plasma insulin concentrations compared to CkC (P < 0.05 for each) (Fig. 1c). The higher insulin levels in DbE were associated with an increase in pancreas weight compared to CkC and DbC (Fig. 1d). To evaluate urine glucose excretion (UGE) at the end of the study, we normalized urine glucose concentration to fasting glucose concentration and observed that the UGE of DbC and DbE mice were 54- and 111-fold higher than that of CkC (Fig. 1e; P < 0.001).

### BP and BP dipping status is unaffected by SGLT2i

**Fig. 2:**
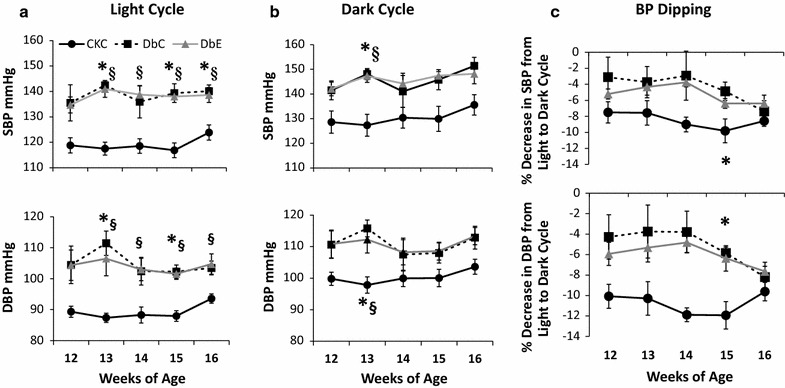
Mean ambulatory systolic and diastolic blood pressures were recorded at weekly intervals during the light **a **and dark **b **cycles. Compared to CkC, both DbC and DbE exhibited a mild elevation in blood pressure (P < 0.05), especially during the light cycle and tended to have impaired blood pressure dipping (**c**), neither of which was affected by empagliflozin treatment. *P < 0.05DbC vs CkC and ^§^P < 0.05 DbE vs CkC

At the end of the treatment period daytime ambulatory systolic BP (SBP) and diastolic BP (DBP) were elevated in both DbC and DbE, compared to CkC (Fig. 2a). SBP and DBP were also elevated during the dark cycle although the trends were not significant over as many time points as occurred during the light cycle (Fig. 2b). The percentage decrease in SBP between the light and dark cycles, also known as BP dipping status, ranged between 7.5 and 9.8% in CkC over study period (Fig. 2c). It should be noted that normal BP dipping is defined as a decrease in BP of at least 10% between the light and dark cycles. BP dipping status tended to be similarly impaired in DbC and DbE between 12 and 15 weeks of age, although all three groups of mice had similar dipping status at 16 weeks of age.

### Cardiac function is improved with SGLT2i

#### Pre-treatment

Compared to CkC, DbC and DbE exhibited impairments in diastolic relaxation (Table 2), including reductions in early septal annular velocity (E′) and the ratio of early (E′) to late (A′) septal annular velocity. This occurred in association with an increase in an index of LV filling pressure, the E/E′ ratio, specifically, the ratio of peak early mitral inflow velocity to peak early septal annular velocity. Left atrial (LA) diameter relative to the diameter of the aorta (Ao) increases in response to an increase in LV filling pressure. The observed increase in the ratio of LA to Ao diameters is consistent with increased LV filling pressure (E/E′ ratio) in both groups of the db/db mice. In contrast, we did not observe differences among groups in early (E) or late (A) mitral inflow velocity or in the E/A ratio, the latter being an often reported marker of diastolic dysfunction. There were no significant differences in the systolic function parameters, ejection fraction (EF) and fractional shortening (FS) among the three groups. As expected in the setting of obesity, cardiac output (CO) and stroke volume (SV) were elevated in the DbC and DbE groups compared to the lean CkC mice. Measures of LV wall thickness tended to be slightly greater in DbC and DbE and LV lumen diameters were similar among groups. Relative wall thickness did not vary significantly among the three groups of mice.

**Table 2:** Summary (mean ± SE) of cardiac pulse wave, tissue Doppler and M-mode imaging parameters measured before (pre) and after treatment (post) with the SGLT-2 inhibitor

Parameter	Pre-treatment				Post-treatment			
	CkC (9)	DbC (6)	DbE (6)	P value	CkC (9)	DbC (6)	DbE (6)	P value
Diastolic function								
E, m s^−1^	665 ± 28	641 ± 40	696 ± 50	ND	632 ± 25	680 ± 29	647 ± 57	ND
A, m s^−1^	455 ± 34	508 ± 19	463 ± 34	ND	417 ± 24	510 ± 31	470 ± 45	ND
E/A	1.52 ± 0.12	1.26 ± 0.05	1.53 ± 0.12	ND	1.5 ± 0.08	1.35 ± 0.09	1.39 ± 0.06	ND
Dt, ms	29.4 ± 3.0	32.7 ± 3.9	27.8 ± 1.8	ND	24.9 ± 1.8	31.1 ± 3.3	26.3 ± 1.8	ND
E′, m s^−1^	27.0 ± 1.8	14.0 ± 1.6*	15.9 ± 2.2	<0.001	26.9 ± 1.4	15.5 ± 1.7*	20.4 ± 1.2	<0.001
A′, m s^−1^	21.5 ± 2.5	16.6 ± 0.8	18.0 ± 4.1	ND	19.0 ± 1.7	18.3 ± 2.7	11.9 ± 0.9^§^	0.036
E′/A′	1.31 ± 0.09	0.84 ± 0.08*	0.93 ± 0.07^§^	0.002	1.47 ± 0.11	0.89 ± 0.08*^,†^	1.76 ± 0.17	<0.001
E/E′	25.3 ± 1.7	48 ± 5.6*	47 ± 2.5^§^	<0.001	24 ± 1.6	46 ± 4.4*^,†^	29 ± 1.8	<0.001
(E/E′/IDd)	8.7 ± 0.4	13.4 ± 1.8	12.4 ± 0.6	ND	7.8 ± 0.5	12.3 ± 1.3*^,†^	7.8 ± 0.9	0.009
IVRT, ms	20.9 ± 1.8	19.9 ± 0.8	18.0 ± 1.0	ND	19.0 ± 2.3	16.0 ± 0.4	15.8 ± 0.4	ND
LA, mm	1.55 ± 0.10	2.35 ± 0.13*	2.25 ± 0.10^§^	<0.001	1.40 ± 0.06	1.97 ± 0.03*	2.04 ± 0.1^§^	<0.001
Ao, mm	1.21 ± 0.06	1.37 ± 0.02	1.42 ± 0.05	0.077	1.26 ± 0.06	1.29 ± 0.02	1.27 ± 0.05	ND
LA/Ao	1.29 ± 0.07	1.72 ± 0.09*	1.60 ± 0.11^a^	0.009	1.14 ± 0.08	1.54 ± 0.11*	1.61 ± 0.1^§^	0.003

Parameter	Pre-treatment				Post-treatment			
	CkC (5)	DbC (6)	DbE (6)	P value	CkC (5)	DbC (6)	DbE (6)	P value
Systolic function								
EF, %	72 ± 6	76 ± 1	71 ± 2	ND	74 ± 2	74 ± 3	76 ± 2	ND
FS, %	42 ± 5	44 ± 1	39 ± 2	ND	42 ± 2	43 ± 3	45 ± 2	ND
CO, ml min^−1^	21 ± 2	41 ± 5*	41 ± 4^§^	0.001	29 ± 3	49 ± 5*	39 ± 5	0.02
SV, μl	56 ± 4	101 ± 11*	95 ± 5^§^	0.016	63 ± 7	108 ± 9*	87 ± 10	0.01
Left ventricle wall and lumen measures								
LVAWTd, mm	0.70 ± 0.07	0.91 ± 0.05*	0.85 ± 0.04	0.052	0.81 ± 0.05	0.82 ± 0.11	0.84 ± 0.05	ND
LVIDd, mm	3.40 ± 0.14	3.52 ± 0.11	3.68 ± 0.14	ND	3.50 ± 0.05	3.79 ± 0.17	3.86 ± 0.19	ND
LVPWTd, mm	0.69 ± 0.04	0.83 ± 0.04*	0.78 ± 0.04	0.026	0.75 ± 0.02	0.92 ± 0.09	0.83 ± 0.05	ND
LVIDs, mm	2.00 ± 0.24	1.96 ± 0.08	2.23 ± 0.13	ND	2.13 ± 0.12	2.28 ± 0.17	2.27 ± 0.11	ND
RWT	0.41 ± 0.03	0.49 ± 0.03	0.45 ± 0.03	ND	0.45 ± 0.02	0.48 ± 0.08	0.43 ± 0.03	ND
LVM, mg	59 ± 3	86 ± 4*	81 ± 3^§^	0.001	76 ± 3	97 ± 7	98 ± 9	0.073
LVM/TL, mg mm^−1^					4.30 ± 0.17	5.56 ± 0.36	5.71 ± 0.52	0.057

*E* velocity of early mitral flow, *A* velocity of late mitral flow, *Dt* deceleration time of early mitral inflow, *E′* early peak velocity of septal annulus, *A′* late peak velocity of septal annulus, *E/E′* index of LA filling pressure, *(E/E′)/LVIDd* index of diastolic stiffness, *IVRT* isovolumic relaxation time, *LA* left atrium diameter, *Ao* aorta diameter, *EF* ejection fraction, *FS* fractional shortening, *CO* cardiac output, *SV* stroke volume, *LVAWTd* anterior wall thickness at end diastole, *LVIDd* LV inner diameter at end diastole, *LVPWTd* LV wall thickness at end diastole, *LVIDs* LV inner diameter at end systole, *RWT* relative wall thickness, *LVM* LV mass, *TL* tibia length (only available post-treatment), *ND* no differences

Numbers in parentheses are sample sizes. Within time point post hoc comparisons: * P < 0.05 for CkC vs DbC; ^†^ DbC vs DbE; ^§^ CkC vs DbE; ^a^ P < 0.051CkC vs DbE

#### End of treatment

**Fig. 3:**
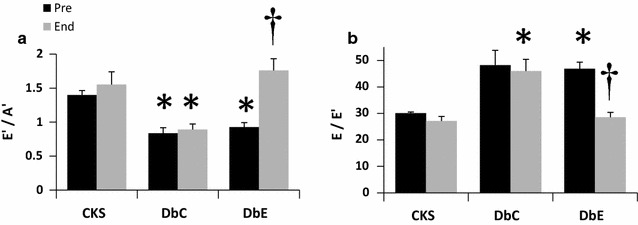
Echocardiographic assessment of diastolic function. Cardiac function was evaluated in 11 week old mice prior to treatment (Pre) and again at 15 weeks of age at the end of treatment. Bar graphs show significant improvements in diastolic parameters in DbE compared to DbC including the **a** Tissue Doppler derived E′/A′ ratio indicating improved septal wall motion and **b** E/E′ ratio indicating improved LV filling pressure. *P < 0.05 compared to CKC at the same time point; ^†^P < 0.05 compared to DbC at the same time point

The diastolic impairments observed in the untreated DbC group at the earlier time point were, in most cases, maintained until the end of the study (Table 2; Fig. 3). On the other hand, early septal annular velocity (E′) tended to increase and late septal annular velocity (A′) tended to decrease in DbE relative to measurements taken 5 weeks earlier. These modest improvements in E′ and A′ contributed to the significant increase in the E′/A′ ratio in DbE (Fig. 3a). Moreover, LV filling pressure (E/E′) was significantly lower in DbE compared to DbC, yet this was not accompanied by a decrease in the LA/Ao ratio (Fig. 3b). This suggests that reversal of LA remodeling may not occur contemporaneously with therapeutic reduction in filling pressure [37]. In contrast, we did not observe differences among groups in early (E) or late (A) mitral inflow velocity or in the E/A ratio, nor were there differences in indices EF or FS. Relative to CkC, CO and SV were still significantly elevated in the DbC, but not DbE mice.

### LV hypertrophy is ameliorated by SGLT2i

**Fig. 4:**
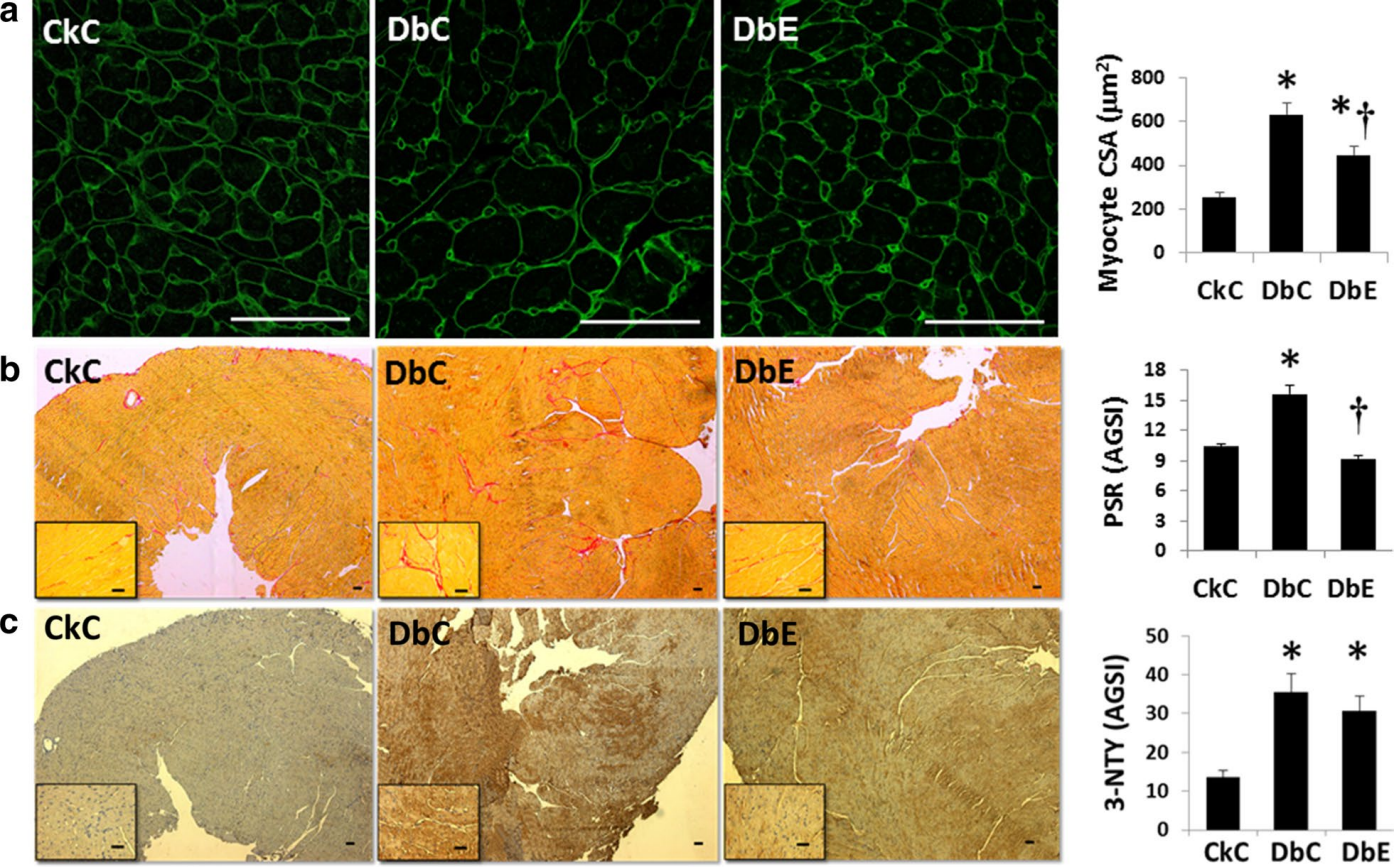
Myocardial remodeling, but not oxidative stress is improved with empagliflozin. **a** WGA staining for determination of myocyte cross sectional area (CSA), magnification ×40, **b** Picrosirius red staining for determination of interstitial fibrosis. Magnification ×5, *inset* ×40 and **c** 3-nitrotyrosine immunostaining for determination of oxidative stress. Magnification ×40, *inset* ×40. *P < 0.05 vs CkC and ^†^P < 0.05 vs DbC. *AGSI* average grey scale intensities. All *scale bars* 50 μm

We observed increases in LV mass, determined by M-mode ultrasound, in DbC and DbE compared to CkC (Table 2). On the other hand, echocardiographic measures of LV wall thickness at end diastole (LVAWTd and LVPWTd) and lumen diameters at end diastole and end systole (LVIDd and LVIDs) were similar among the three groups of mice. Left ventricular cardiomyocyte cross sectional area, a correlate of cardiomyocyte hypertrophy, was elevated in both DbC compared to CkC (Fig. 4a). Interestingly, cardiomyocyte cross sectional area was significantly reduced in DbE compared to DbC, although it did not reduce to that of CkC. The increases in CO, LV filling pressure, cardiomyocyte size and LV mass, in the absence of an increase in LV wall thickness, comprise a suite of traits suggestive of eccentric hypertrophy. It should be noted that the most prevalent LV remodeling abnormality in obese hypertensive individuals is eccentric hypertrophy [38]. The lower CO and LV filling pressure observed in DbE are consistent with reduced cardiomyocyte cross sectional area and suggest that the progression of the hypertrophic phenotype is mildly ameliorated with EMPA treatment.

### Myocardial fibrosis is improved with SGLT2i

At the end of treatment, the myocardium of DbC exhibited 50% greater picrosirius red staining than that of CkC, indicative of increased collagen I and III deposition in the interstitium (Fig. 4b). Myocardial picrosirius red staining in DbE was significantly reduced compared to DbC and was not different from CkC. Immunostaining for the more tensile type 1 collagen revealed a similar pattern of increasing collagen 1 in DbC compared to both CkC and DbE (14.8 ± 3.3 vs 11.1 ± 0.9 and 10.2 ± 0.3, respectively; P > 0.05), however the differences were not statistically different.

### Myocardial oxidative/nitrosative stress, AGE and RAGE

**Fig. 5:**
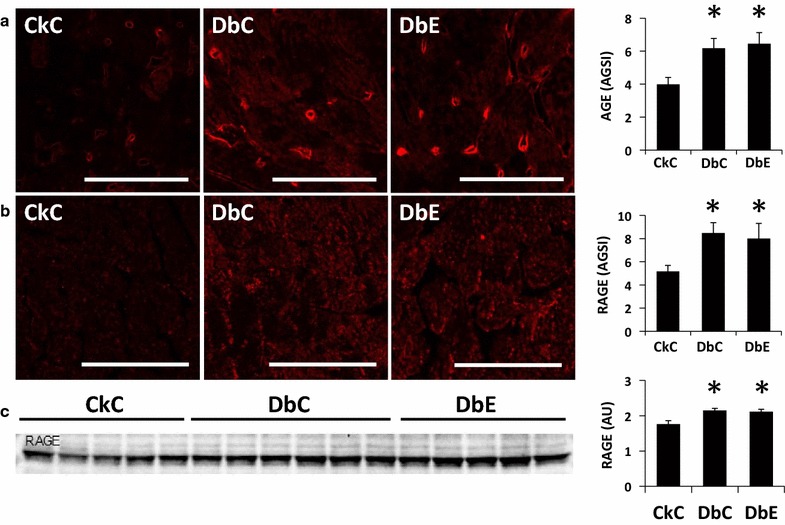
Five weeks of empagliflozin treatment reduces the profibrotic protein expression levels of **a**, **b** ENaC and **c** SGK1. *AGSI* average grey scale intensities, *AU* arbitrary units *P < 0.05 vs CkC; ^†^P < 0.05 vs DbC. *Scale bars* 50 μm

Oxidant stress contributes to AGE/RAGE activation in diabetes. The myocardium of DbC exhibited 2.6-fold greater 3-NTY immunostaining compared to CkC, indicative of increased accumulation of nitro tyrosinolated protein damage in the interstitium (Fig. 4c). Compared to DbC, staining of 3-NTY tended to be slightly lower in DbE, compared to DbC, but was not significant (P > 0.05). In DbC, there were concomitant increases of 55 and 66%, respectively, in myocardial expression of AGE and RAGE, compared to CkC (Fig. 5a–c). In the myocardium of CkC, the basal level of AGE staining indicates low level expression in the parenchyma with more prominent staining in the vascular wall. In the DbC, there are increases in AGE staining in both the parenchyma and vascular wall. EMPA did not reduce the accumulations of AGE or RAGE in the DbE heart. Although oxidant damage likely contributes to fibrosis and diastolic dysfunction, we and others have observed interventional improvement in diastolic dysfunction in the absence of significant reductions in myocardial oxidative/nitrosative stress [31, 39].

**Fig. 6:**
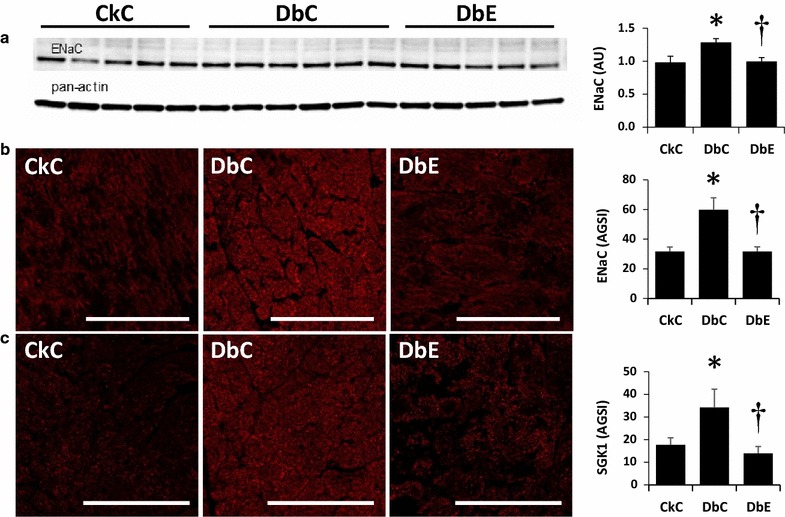
Empagliflozin reduces myocardial profibrotic signaling. Five weeks of empagliflozin treatment reduces the profibrotic proein expression levels of **a, b **ENaC and **c **SGK1. *AGSI* average grey scale intensities, *AU* arbitrary units *P < 0.05 vs CkC; P < 0.05 vs DbC. *Scale bars* 50 µm

### SGK1 and ENaC expression are reduced with SGLT2i

SGK1 is highly expressed in the myocardium, especially in the setting of obesity and diabetes and is emerging as a contributor to cardiac fibrosis/stiffening, hypertrophy and impaired cardiac relaxation [7–9]. Additionally, SGK1 upregulates ion channels, including ENaC, and is likewise a potent inducer of the SGLT1, a novel cardiac glucose transporter in type 2 diabetes [40]. Myocardial ENaC protein level (Fig. 6a), assessed by immunoblotting, was significantly elevated in DbC, compared to both CkC and DbE. We examined the expression of the α-subunit of the ENaC, as well as SGK1 in the LV using immunofluorescence. In this study, we observed ~2-fold increases in myocardial expression of both ENaC and SGK1 in DbC compared to CkC, and normalization of these protein levels in DbE (Fig. 6b,c). In addition, myocardial ENaC protein level, assessed by immunoblotting, was significantly elevated in DbC, compared to both CkC and DbE. We further examined signaling pathways for hypertrophy comprising Akt and ERK activation. Although the changes in phospho Akt ser473 and Thr308 were not statistically different between the three groups, the ratio of phospho Akt to total Akt was moderately increased (P < 0.055 for ser473 and P < 0.079 for thr308) due to significant decrease in total Akt in DbC and DbE groups compared to CkC (Additional file 1: Figure S2). Activation of ERK1/2 is another signaling pathway implicated in cardiac hypertrophy, but this pathway was not affected either in DbC and DbE (Additional file 1: Figure S2).

### Myocardial ultrastructure

**Fig. 7:**
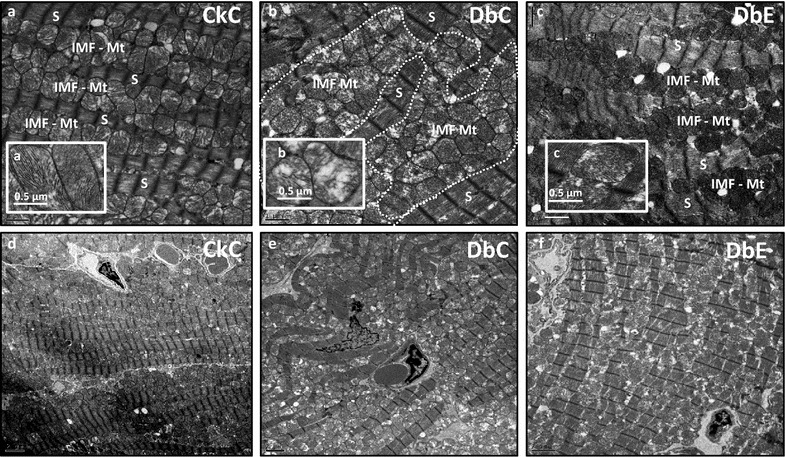
Mitochondrial expansion and sarcomere disorganization in DbC is improved by empagliflozin treatment. **a** Illustrates the normal appearance of the inter myofibrillar (IMF) mitochondria (Mt) in the myocardium of CkC. Note the orderly row of sarcomeres (S) alternating with a row of IMF-Mt. *Inset* **a** shows normal cristae structure. **b** Depicts the marked IMF-Mt expansion with attenuation of the Mt matrix electron density, loss of Mt cristae (*inset* **b**), fusion of Mt cristae and Mt fragmentation in the diabetic DbC models. Also note the disorganized appearance of the sarcomeres. **c** Demonstrates that empagliflozin protects the cardiomyocyte from IMF-Mt expansion, decreased IMF-Mt matrix electron density, IMF-Mt cristae fragmentation (*inset* **c**), fusion of cristae and loss and sarcomere disorganization. Magnification ×2000; *bar* 1 µm in **a**–**c**. *Insets* **a**–**c** depict Mt cristae structure. Magnification ×4000; *bar* 0.5 µm. **d**–**f** represent CkC, DbC and DbE, respectively, at lower magnifications ×800; *bar* 2 µm

Compared to CkC, DbC exhibited a marked expansion of inter myofibrillar mitochondria (IMF Mt) resulting in a disorganized appearance of sarcomeres (Fig. 7). Additionally, mitochondria displayed attenuation of matrix electron density, loss of cristae, fusion of cristae and mitochondrial fragmentation and these ultrastructural anomalies were improved by EMPA treatment.

## Discussion

Collectively, the results of this investigation support the hypothesis that treatment with the SGLT2i, EMPA, improves cardiac diastolic function in female mice in the setting of obesity and diabetes, even in the absence of a reduction in BP. The improvement in diastolic function was associated, not only with improved glycemia, but with improvements in cardiac structure, including reductions in interstitial myocardial fibrosis and associated pro-fibrotic SGK1/ENaC protein expression levels, cardiomyocyte hypertrophy and cardiomyocyte mitochondrial ultrastructure. Although EMPA is reported to reduce BP in diabetic humans [41], we did not observe lower BP in treated mice, nor did we see an improvement in BP dipping status compared to diabetic mice not receiving EMPA. Thus, functional and structural improvements in the heart of EMPA-treated mice occurred in the absence of BP reduction.

Administration of SGLT2 inhibitors is reported to improve glycemia and promote weight loss and body fat mass reduction in humans and rodents with diabetes, in part, due to caloric loss associated with increased urinary glucose excretion and a metabolic shift from carbohydrate to fatty acid oxidation [42, 43]. In this regard, a 4 week course of EMPA led to a slight reduction in triglycerides in patients with type 2 diabetes [44]. By the end of this study, we observed that DbC mice exhibited moderate glycosuria indicated by the 54-fold increase in urinary glucose excretion compared to CkC. Importantly, at the end of the study period, EMPA-treated db/db mice (10 mg kg^−1^ day^−1^) exhibited twice the urine glucose excretion of untreated db/db mice, thus validating long-term SGLT2 inhibition. Pharmacologic inhibition of SGLT2 has been reported to induce increases in plasma insulin concentration in db/db mice [45] and Zucker diabetic fatty rats [46] in association with preservation of β-cell insulin secretion and consequent improved glycemia. Consistent with those previous animal studies, we observed increased plasma insulin concentration. Our results demonstrate that the low dose of EMPA used in this study blunted the progression of hyperglycemia by inducing glycosuria. Improved glycemia is associated with preservation of β-cell function [47, 48]. Thus, the higher concentration of plasma insulin we observed with EMPA administration reflects therapeutic preservation of β-cell function and a slower decline in plasma insulin concentration. We did not observe differences in body weight or composition (lean or fat mass) or plasma or liver triglycerides between DbC and DbE. Similarly, treatment of female db/db mice for 8 weeks of EMPA at the same dose as that used herein in our study had no effect on body weight [13].

In this study we have explored the mechanisms underlying diastolic dysfunction in female db/db mice, including myocardial fibrosis, hypertrophy and calcium handling. A major determinant of impairment of the passive properties of diastolic relaxation is cardiac fibrosis. In the setting of obesity and diabetes, an increase in myocardial interstitial fibrosis can occur in response to increased pressure or volume loading conditions and this is thought to impair normal rapid ventricular relaxation. Female diabetic db/db mice exhibit increased cardiac interstitial fibrosis as early as 8 weeks of age [49] and we observed increased fibrosis in 16 week old females (Fig. 4b). Although such a pathological remodeling response reduces LV wall stress and may confer short-term benefits, stiffening of the myocardium can lead to reduced myocardial wall velocity during early diastole and more reliance on atrial contraction to complete filling of the LV in late diastole. Impairment in ventricular wall relaxation can be evaluated during routine echocardiography using Tissue Doppler Imaging (TDI) which allows for determination of the peak velocities of the myocardial wall during early (E′) and late diastole (A′). Indeed, our TDI examination revealed a reduction in the E′/A′ ratio in DbC mice before treatment began and at the end of the study suggesting chronic impaired LV wall relaxation early in diastole and enhanced late filling. Although at baseline this impairment existed in the DbE group by the end of treatment period this impairment was resolved. Perhaps most importantly, were the differences in LV filling pressure or the E to E′ ratio, among groups of mice. Unlike some diastolic parameters, such as the E/A ratio, E/E′ is a load-independent parameter. DbC exhibited increased LV filling pressure at baseline, as well as at the end of the study period, whereas, DbE mice had elevated LV filling pressure at baseline and this impairment was normalized by the end of the study. The decrease in LV filling pressure and reduction in interstitial fibrosis are consistent with an improvement in diastolic function with EMPA treatment. Our findings of impairments in TDI derived diastolic function parameters are also consistent with a recent preliminary analysis from the EMPA-REG OUTCOME trial [50]. In that study baseline echocardiography was performed on diabetes patients followed by treatment with 10 mg day^−1^ EMPA for 3 months and follow up echocardiography. EMPA resulted in an increase in E′ and a decrease in the LV mass index. These interesting findings suggesting that EMPA improves diastolic function will likely be explored more extensively in ongoing clinical trials.

Like cardiac fibrosis, left ventricular hypertrophy, a frequent correlate of diastolic dysfunction [51], occurs in response to increased pressure or volume loading conditions common in the setting of obesity and diabetes. We observed that standard hypertrophic parameters, including LV mass, LV mass adjusted for tibia length and cardiomyocyte cross sectional area, were increased or tended to be increased in both DbC and DbE compared to CkC, and these results are consistent with a hypertrophic phenotype. Moreover, others have reported increased LV mass and wall thickness in female diabetic db/db mice of similar age and that remodeling was associated with diastolic dysfunction [20]. In this study, relative wall thickness (RWT) did not differ among the three groups of mice. Prior to the start of treatment, anterior (LVAWTd) and posterior wall (LVPWTd) thicknesses at end diastole were moderately increased in DbC compared to CkC (P < 0.5). Wall thickness were not different between DbC and DbE and they tended to be increased in DbE compared to CkC (P > 0.05). Moreover, there were no differences in RWT between the groups at the end of the study. Therefore, the increase in LV mass, in concert with no change in RWT, suggests that the LV of DbC exhibited eccentric, rather than concentric, hypertrophy. We speculate that cardiomyocytes may be lengthening in response to volume overload associated with profound obesity, as evidenced by the increase in CO in DbC. Nonetheless, DbC cardiomyocytes were more hypertrophied than those of DbE and CkC and this may be the result of stress on the LV wall caused by an increase in LV filling pressure. DbE showed lesser cardiomyocyte hypertrophy likely in response to a reduction in LV filling pressure and the decrease in cardiomyocyte hypertrophy was only a partial effect compared to the normalizing effect of EMPA on interstitial fibrosis. This suggests cardiac fibrosis contributes mainly to diastolic dysfunction.

We explored several pathways that contribute to cardiac remodeling and diastolic dysfunction, some of which have not been examined in previous studies. Emerging evidence supports a role for SGK1 and ENaC in promoting fibrosis and adverse hypertrophy in human and murine heart disease [7, 8]. SGK1 is highly expressed in human and murine hearts and is upregulated in pathophysiological settings, including obesity, heart disease and diabetes [40]. SGK1 regulates the expression of a number of ion channels, including ENaC [7, 9] which is upregulated in tissues in the setting of obesity and diabetes [52]. Indeed, SGK1 is rapidly activated and induces adverse ventricular remodeling, including, fibrosis, an increase in cardiomyocyte cell size and LV hypertrophy in a murine model of transthoracic aortic constriction [7, 8]. On the other hand, SGK1 may protect cardiomyocytes from apoptosis [8]. Little is known about the role of SGK1 and ENaC in the diabetic db/db heart or the impact of EMPA on myocardial ENaC and SGK1 expression/activation. To our knowledge, this is the first study to examine the expression of SGK1 and the α-subunit of the epithelial sodium channel, ENaC, in the parenchyma and vasculature of the LV. In this study, we observed increases in myocardial expression of SGK1 and ENaC and normalization of these protein levels by EMPA. These results suggest that the improvement in cardiac function may be modulated, in part, through improvement in SGK1/ENaC activity in the heart. The changes in expression of these proteins may be due to significant improvement in hyperglycemia since high glucose has been shown to increase the expression of SGK1 and ENaC in distal renal tubular cells in vitro [53]. However, the effects of high glucose on SGK1 and ENaC in cardiomyocytes or coronary endothelial cells of db/db mice has not been examined. We also examined the potential contribution of the pro-hypertrophic signaling pathways, Akt and ERK (Additional file 1: Figure S2). Although compared to CkC, Akt tended to be activated in both DbC and DbE, there was no difference in the activation state between DbC and DbE. The activation state of ERK did not differ among groups of mice. Thus, the modest improvement in hypertrophy in DbE is not likely mediated by effects of EMPA on Akt or ERK. The absence of evidence for contributions from Akt and ERK to cardiac remodeling in female db/db mice examined in this study highlight the potential contributions of SGK1 and eNAC to cardiac fibrosis and hypertrophy.

In this study, changes in sarco/endoplasmic reticulum Ca^2+^-ATPase (SERCA), SERCA to phospholamban (PLB) ratio and phosphorylation of PLB may potentially affect diastolic function [54]. Although others have reported altered gene expression of SERCA2A and PLB in 18 week old female db/db mice, we observed no differences among groups in protein expression (not shown) and thus cannot ascribe the differences in diastolic function observed among these proteins. Thus, we conclude these pathways do not explain the improvements in cardiac function observed. On the other hand, we did observe improved cardiomyocyte mitochondrial ultrastructure and sarcomere organization that may, in part, contribute to improved diastolic function as we have observed in this and other rodent models of cardiac dysfunction and obesity [29, 31, 32, 55].

Signaling through RAGE has been implicated in numerous disease conditions, including diabetes and heart disease. RAGE can bind multiple ligand types, including, but not limited to a heterogenous group of advanced glycation end products (AGEs). AGEs accumulate as a consequence of long-term hyperglycemia and contribute to the pathogenesis of diabetic cardiomyopathy [56]. AGE accumulation can lead to generation of reactive oxygen species (ROS) and upregulation of RAGE. Moreover, AGEs have been shown to induce the expression of ENaC via activation of SGK1 [57]. Recent evidence in mice suggests there is RAGE upregulation in cardiomyocytes that contributes to cardiomyopathy [58]. Herein, we show that myocardial AGE expression is increased in DbC and DbE, compared to CkC, and observe that it is widely distributed throughout the LV wall, including in the vasculature. This occurred in concert with increased RAGE expression. In the DbC, the increases in both AGE and RAGE were associated with an increase in oxidative stress. Nonetheless, despite the modest improvement in HbA1c in DbE, EMPA did not reduce AGE accumulation in the heart. In this regard, a recent study utilizing a rodent model of type 1 (streptozotocin model) diabetes, reported that a low dose of EMPA (10 mg kg^−1^ day^−1^), the same dose used in this study, was not associated with suppression of AGE, RAGE or ROS formation in aortic tissue or serum methylglyoxal, an AGE precursor, although treatment did improve aortic remodeling and reactivity to acetylcholine [16]. In that study, a threefold higher dose of EMPA did reduce AGE and RAGE expression, as well as ROS formation in the aortic wall. It should be noted that the glycation process in vivo results in formation of early glycation products, such as HbA1c and glycated albumin, whereas advanced glycation products require longer times to accumulate relative to early glycation products. In this regard, a previous study reported that advanced glycated Hb (Hb-AGE) was significantly increased with high level of HbA1c in diabetic patients, but Hb-AGE did not correlate with diabetes duration and correlated poorly in a well-controlled sub-group [59].

Evidence from a recent clinical trial (EMPA-REG BP™) examining the effects of a 12 week course of EMPA on male and female patients with type 2 diabetes and hypertension demonstrates improvements in both sexes in SBP and DBP (~3.9 and 1.5 mmHg, respectively compared to placebo), as well as indirect markers of arterial stiffness and vascular resistance [41, 60]. In the untreated db/db mice examined here (DbC), we observed increased SBP and DBP, as well as impaired BP dipping, all of which were unaffected by EMPA. It is possible that the 5 week duration of our study was too brief to induce a detectable decrease in BP.

Coinciding with the preparation of this manuscript there was a more recent report from the EMPA-REG outcome trial demonstrating improvement in Tissue Doppler derived diastolic function in diabetes patients administered EMPA [50]. That EMPA resulted in similar improvement in myocardial wall relaxation in both diabetes patients and in diabetic db/db mice suggests that the present db/db model has potential clinical translational relevance.

The absence of data on the effects of EMPA on myocardial metabolism and insulin sensitivity is a limitation of this study. In this regard, a recent study reported increases in glucose disposal rate and liver, kidney and heart tissue glucose uptake, by euglycemic–hyperinsulinemic clamp, in female db/db mice treated with EMPA for 8 weeks at the same dose used in this study [13]. Thus it is possible that the improvement in diastolic dysfunction observed in this study could be due, in part, to increased myocardial glucose uptake and improvement in myocardial insulin sensitivity. Further study is needed to determine whether improvement in myocardial insulin sensitivity contributes to improved diastolic function in db/db mice treated with EMPA.

In conclusion, data presented herein support a newly described pleiotropic protective affect of EMPA on diastolic function and cardiac structure in the diabetic db/db mouse with established impaired diastolic relaxation, albuminuria and elevated BP. Specifically, we observed improvement in diastolic function, myocardial fibrosis, cardiomyocyte hypertrophy and ultrastructure of inter myofibrillar mitochondria that were associated with a significant improvement in glycemia, as well as improvement in myocardial expression of SGK1 and ENaC. Despite the improvement in glycemic control, HbA1c and fasting glucose were still elevated above normoglycemia values. Moreover, we did not see any marked improvements in body composition, lipid (plasma or liver triglycerides) or BP control or BP dipping, or reductions in myocardial accumulation of AGE/RAGE and protein nitrosylation with 5 weeks of EMPA treatment. It was recently highlighted that the unexplained aspects of the EMPA-REG OUTCOME results are that the cardiovascular and kidney benefits of EMPA occurred without dramatic improvements in glycemic, lipid, or BP control [61]. Therefore, it is possible that the pleiotropic effects of EMPA relate to factors other than improvements in glycemia and lipidemia. In this regard, the effect of EMPA on metabolic remodeling in tissues is emerging [61, 62]. Additional studies are needed to further elucidate the potential role for myocardial SGK1 and ENaC as mediators of the efficacy of EMPA. The findings of this investigation in a preclinical model suggest a potential clinical utility for EMPA in the treatment of diastolic dysfunction given the high incidence of diastolic dysfunction and the cardiovascular risks associated with this abnormality in the diabetic population and in women in particular.

## Additional file

**Additional file 1.** Additional figures.
