# Supplementary material for: Sodium glucose transporter 2 (SGLT2) inhibition with empagliflozin improves cardiac diastolic function in a female rodent model of diabetes
Source: Cardiovasc Diabetol. 2017 Jan 13;16:9. doi: 10.1186/s12933-016-0489-z (PMC5237274; doi:10.1186/s12933-016-0489-z)
Supplement: Supplementary file 1 — Additional file 1. Additional figures. [file 12933_2016_489_MOESM1_ESM.docx]

**Sodium Glucose Transporter 2 (SGLT2) Inhibition with Empagliflozin improves Cardiac Diastolic Function in a Female Rodent Model of Diabetes**

*Javad Habibi^1,4^, *Annayya R. Aroor^1,4^, James R. Sowers^1,3,4,5^, Guanghong Jia^1,4^, Melvin R. Hayden^1^ , Mona Garro^1,4^, Brady Barron^1,4^, Eric Mayoux^7^, Scott R. Rector^4,6^, Adam Whaley-Connell^1,2,4^ and Vincent G. DeMarco^1,3,4^

Department of Medicine, Division of Endocrinology, Diabetes and Cardiovascular Center^1^ and and Division of Nephrology^2^, Department of Medical Pharmacology and Physiology^3^, University of Missouri, School of Medicine, Research Service, Harry S. Truman Memorial Veterans Hospital^4^, the Dalton Cardiovascular Research Center^5^, Departments of Medicine-Gastroenterology and Hepatology and Nutrition and Exercise Physiology^6^, Columbia, MO, USA and Department of Cardiometabolic Diseases Research, Boehringer-Ingelheim^7^, Biberach, Germany

* These authors contributed equally to this work.

**Running Title:** Empagliflozin improves diastolic function

**Corresponding Author:**

Dr Vincent G. DeMarco, PhD

Department of Internal Medicine

Division of Endocrinology, Diabetes and Metabolism

University of Missouri-Columbia School of Medicine

One Hospital Drive

Columbia, MO 65212

Phone: (573) 814-6000 ext 53678

E-mail: demarcov@missouri.edu

**Key Words:** Empagliflozin, SGLT2, cardiac function

**Sources of Funding**: This work was supported by an unrestricted research grant from Boehringer Ingelheim Pharma (VGD), as well as support from the National Institutes of Health (R01-HL073101 RO1-HL107910), VA Merit (###) to JRS, Veteran Affairs (VA) Grants VA-CDA2 BX001299 (RSR) and Boehringer Ingelheim Pharma (EM). This work was supported by resources and facilities at the Harry S. Truman Memorial Veterans’ Hospital in Columbia, MO.

**Disclosures:** VGD received support from Boehringer-Ingelheim, EM is an employee of Boehringer-Ingelheim.

**Online Supplementary Material**


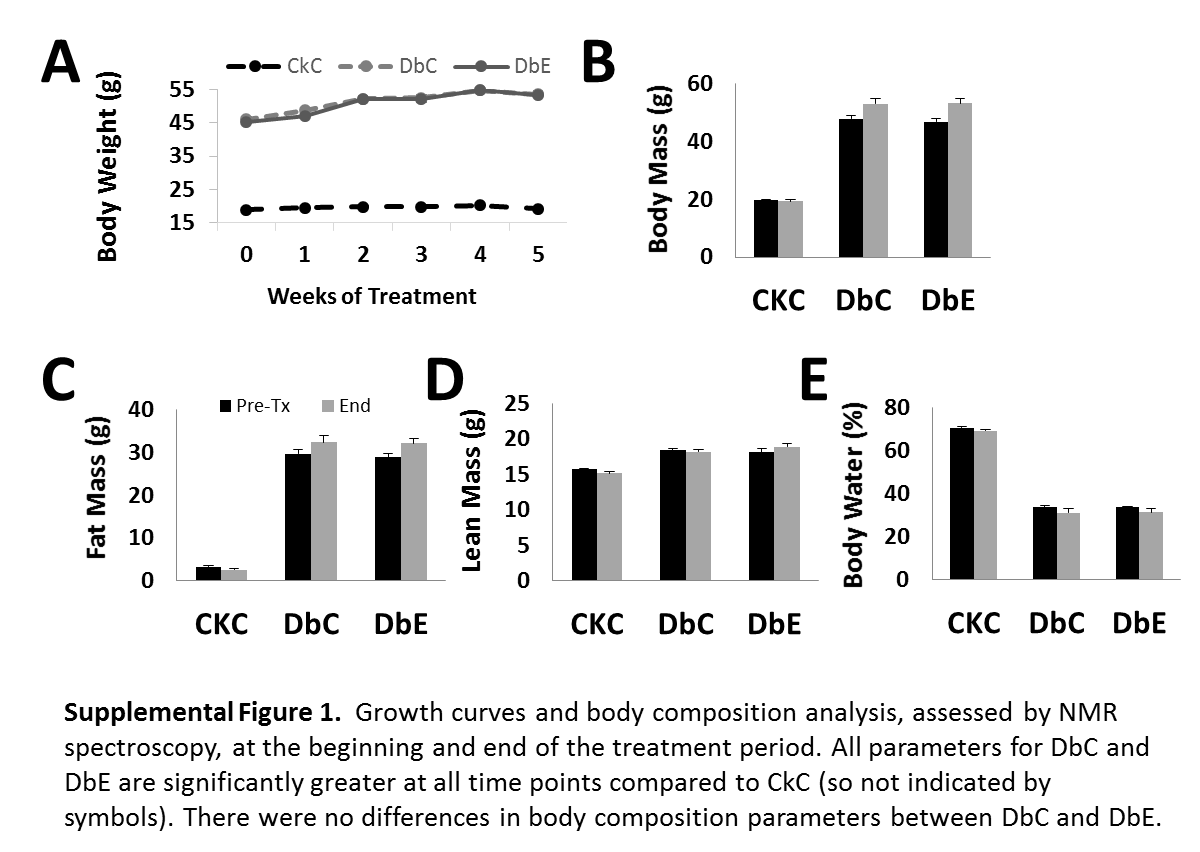


**
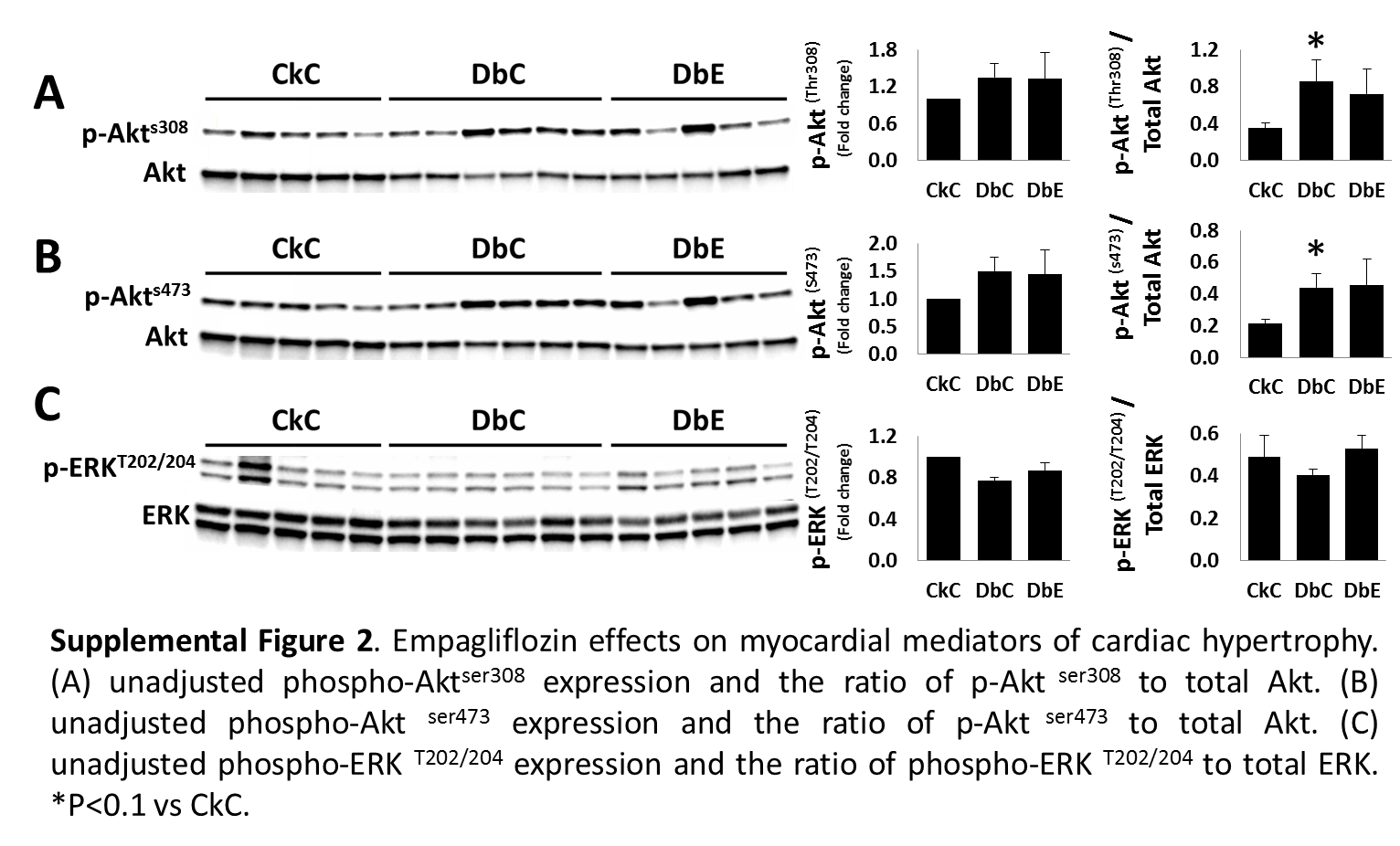
**
